# Supplementary material for: ADAPTS: Automated deconvolution augmentation of profiles for tissue specific cells
Source: PLoS One. 2019 Nov 19;14(11):e0224693. doi: 10.1371/journal.pone.0224693 (PMC6863530; doi:10.1371/journal.pone.0224693)
Supplement: S1 Vig — (HTML) [file pone.0224693.s001.html]

ADAPTS (Automated Deconvolution Augmentation of Profiles for Tissue Specific cells) Vignette #1: Matrix Augmentation


# ADAPTS (Automated Deconvolution Augmentation of Profiles for Tissue Specific cells) Vignette #1: Matrix Augmentation

#### *Samuel Danziger, PhD*

#### *2019-09-20*

Part I - A: Deconvolve purified CD138+ tumor samples using LM22 Note: ADAPTSdata and ADAPTSdata2 come from GitHub devtools::install\_github(“sdanzige/ADAPTSdata”) devtools::install\_github(“sdanzige/ADAPTSdata2”)

```
library(ADAPTS)
library(ADAPTSdata)
library(ADAPTSdata2)
library(preprocessCore)
library(pheatmap)
library(mclust)
#> Package 'mclust' version 5.4.2
#> Type 'citation("mclust")' for citing this R package in publications.
doParallel::registerDoParallel(cores = parallel::detectCores())

set.seed(42)

LM22 <- ADAPTS::LM22
cd138p <- ADAPTSdata::cd138p
wbm <- ADAPTSdata2::wbm
tumorPer <- ADAPTSdata2::tumorPer
LM22.source <- ADAPTSdata2::fullLM22
addMGSM27 <- ADAPTSdata::addMGSM27
```

```
cellCounts <- estCellPercent.DCQ(LM22, cd138p)
tumorPer$LM22.CD138p.tumor <- colSums(cellCounts[c('Plasma.cells','B.cells.memory'),
    match(make.names(tumorPer$celfileRnas),colnames(cellCounts))])

incBool <- !is.na(tumorPer$CD138_Post_Sort) 
curData <- tumorPer[incBool,]
rmse.lm22.cd138p <- sqrt(mean((curData$CD138_Post_Sort - curData$LM22.CD138p.tumor)^2))
ct.lm22.cd138p <- cor.test(curData$CD138_Post_Sort, curData$LM22.CD138p.tumor)
ct.lm22.cd138p.spear <- suppressWarnings(cor.test(curData$CD138_Post_Sort, curData$LM22.CD138p.tumor, method = 'spearman'))
titleStr <- paste0('LM22 Deconvolution of CD138+ Samples:\nRMSE = ', round(rmse.lm22.cd138p), '; Rho = ', round(ct.lm22.cd138p$estimate,2), '; Spear =', round(ct.lm22.cd138p.spear$estimate,2))
plot(curData$CD138_Post_Sort, curData$LM22.CD138p.tumor, main=titleStr, xlab='% Purity', 
     ylab='Deconvolved Tumor %')

#Save the Deconvolution results
deconEst <- list(est.LM22.CD138p=rowMeans(cellCounts, na.rm=TRUE))
```

Part I - B: Deconvolve WBM samples using LM22

```
cellCounts <- estCellPercent.DCQ(LM22, wbm)
tumorPer$LM22.WBM.tumor <- colSums(cellCounts[c('Plasma.cells','B.cells.memory'),
    match(make.names(tumorPer$celfileRnbx),colnames(cellCounts))])

tumorPer$PC_Mean <- rowMeans(tumorPer[,c('PC_AspNum','PC_BmbxNum')], na.rm=TRUE)
incBool <- !is.nan(tumorPer$PC_Mean) & !is.na(tumorPer$LM22.WBM.tumor)
curData <- tumorPer[incBool,] 
rmse.lm22.WBM <- sqrt(mean((curData$PC_Mean - curData$LM22.WBM.tumor)^2))
ct.lm22.WBM <- cor.test(curData$PC_Mean, curData$LM22.WBM.tumor)
ct.lm22.WBM.spear <- suppressWarnings(cor.test(curData$PC_Mean, curData$LM22.WBM.tumor, method = 'spearman'))
titleStr <- paste0('LM22 Deconvolution of WBM Samples:\nRMSE = ', round(rmse.lm22.WBM), '; Rho = ', round(ct.lm22.WBM$estimate,2), '; Spear =', round(ct.lm22.cd138p.spear$estimate,2))
plot(curData$PC_Mean, curData$LM22.WBM.tumor, main=titleStr, xlab='% WBM Tumor', ylab='Deconvolved Tumor %')

#Save the Deconvolution results
deconEst[['est.LM22.WBM']] <- rowMeans(cellCounts, na.rm=TRUE)
```

Test out LM22 and plot the results

```
LM22.source.copy <- LM22.source
colnames(LM22.source.copy) <- gsub('\\.[0-9]+$', '', colnames(LM22.source.copy))
lm22spill <- ADAPTS::spillToConvergence(LM22, LM22.source.copy, plotIt = FALSE, imputNAs=FALSE)
#> Removing 24 that are in sigMatrix but not geneExpr
#> Removing 79 / 523 genes with missing values
initSpill <- t(lm22spill[[2]])
initSpill <- initSpill[,rownames(initSpill)]
initSpill <- cbind(initSpill, data.frame(others=lm22spill[[2]]['others',]))

#One way to add a title to the legend.  It's ugly, just use Inkscape
#From: https://stackoverflow.com/questions/36852101/r-legend-title-or-units-when-using-pheatmap

pheatmap(initSpill, cluster_rows = FALSE, cluster_cols = FALSE, main = 'LM22 Spillover Matrix')
pheatmap(initSpill, cluster_rows = FALSE, cluster_cols = FALSE, main = 'LM22 Spillover Matrix', filename = 'ADAPTS.fig2.pdf', width = 8, height = 8)
```

Now plot the final spillover matrix

```
finalSpill <- t(lm22spill[[length(lm22spill)]])
finalSpill <- finalSpill[,rownames(finalSpill)]
finalSpill <- cbind(finalSpill, data.frame(others=lm22spill[[length(lm22spill)]]['others',]))

#One way to add a title to the legend.  It's ugly, just use Inkscape
#From: https://stackoverflow.com/questions/36852101/r-legend-title-or-units-when-using-pheatmap

pheatmap(finalSpill, main = 'LM22 Spillover Convergence Matrix')
pheatmap(finalSpill, main = 'LM22 Spillover Convergence Matrix', filename = 'ADAPTS.fig3.pdf', width = 8, height = 8)
```

Part II - Build LM22 + ‘MM.plasma.cell’,‘PlasmaMemory’

```
LM22p <- LM22
augData <- addMGSM27[match(rownames(LM22), rownames(addMGSM27)), ]#grepl('PlasmaMemory',colnames(addMGSM27)) | grepl('MM.plasma.cell',colnames(addMGSM27))]
LM22p$PlasmaMemory <- rowSums(augData[,grepl('PlasmaMemory',colnames(augData))], na.rm=TRUE)
LM22p$MM.plasma.cell <- rowSums(augData[,grepl('MM.plasma.cell',colnames(augData))], na.rm=TRUE)
LM22p$osteoclast <- rowSums(augData[,grepl('osteoclast',colnames(augData))], na.rm=TRUE)
LM22p$osteoblast <- rowSums(augData[,grepl('osteoblast',colnames(augData))], na.rm=TRUE)
LM22p$adipocyte <- rowSums(augData[,grepl('adipocyte',colnames(augData))], na.rm=TRUE)
```

Part II - A: Deconvolve purified CD138+ tumor samples using LM22 + ‘MM.plasma.cell’,‘PlasmaMemory’

```
cellCounts <- estCellPercent.DCQ(LM22p, cd138p)
tumorPer$LM22p.CD138p.tumor <- colSums(cellCounts[c('Plasma.cells','B.cells.memory','MM.plasma.cell','PlasmaMemory'),
    match(make.names(tumorPer$celfileRnas),colnames(cellCounts))])

incBool <- !is.na(tumorPer$CD138_Post_Sort)
curData <- tumorPer[incBool,]
rmse.lm22p.cd138p <- sqrt(mean((curData$CD138_Post_Sort - curData$LM22p.CD138p.tumor)^2))
ct.lm22p.cd138p <- cor.test(curData$CD138_Post_Sort, curData$LM22p.CD138p.tumor)
ct.lm22p.cd138p.spear <- suppressWarnings(cor.test(curData$CD138_Post_Sort, curData$LM22p.CD138p.tumor, method='spearman'))
titleStr <- paste0('LM22 + 5 Deconvolution of CD138+ Samples:\nRMSE = ', round(rmse.lm22p.cd138p), '; Rho = ', round(ct.lm22p.cd138p$estimate,2), '; Spear = ', round(ct.lm22p.cd138p.spear$estimate,2))
plot(curData$CD138_Post_Sort, curData$LM22p.CD138p.tumor, main=titleStr, xlab='% Purity', ylab='Deconvolved Tumor %')

#Save the Deconvolution results
deconEst[['est.LM22p5.CD138p']] <- rowMeans(cellCounts, na.rm=TRUE)
```

Part II - B: Deconvolve WBM samples using LM22 + Bone Marrow Cells

```
cellCounts <- estCellPercent.DCQ(LM22p, wbm)
tumorPer$LM22p.WBM.tumor <- colSums(cellCounts[c('Plasma.cells','B.cells.memory','MM.plasma.cell','PlasmaMemory'),
    match(make.names(tumorPer$celfileRnbx),colnames(cellCounts))])

tumorPer$PC_Mean <- rowMeans(tumorPer[,c('PC_AspNum','PC_BmbxNum')], na.rm=TRUE)
incBool <- !is.nan(tumorPer$PC_Mean) & !is.na(tumorPer$LM22p.WBM.tumor)
curData <- tumorPer[incBool,] 
rmse.lm22p.WBM <- sqrt(mean((curData$PC_Mean - curData$LM22p.WBM.tumor)^2))
ct.lm22p.WBM <- cor.test(curData$PC_Mean, curData$LM22p.WBM.tumor)
ct.lm22p.WBM.spear <- suppressWarnings(cor.test(curData$PC_Mean, curData$LM22p.WBM.tumor, method='spearman'))
titleStr <- paste0('LM22 + 5 Deconvolution of WBM Samples:\nRMSE = ', round(rmse.lm22p.WBM), '; Rho = ', round(ct.lm22p.WBM$estimate,2), '; Spear = ', round(ct.lm22p.WBM.spear$estimate,2) )
plot(curData$PC_Mean, curData$LM22p.WBM.tumor, main=titleStr, xlab='% WBM Tumor', ylab='Deconvolved Tumor %')

#Save the Deconvolution results
deconEst[['est.LM22p5.WBM']] <- rowMeans(cellCounts, na.rm=TRUE)
```

Part III: Augmenting an existing signature matrix

```
olGenes <- rownames(LM22.source)[rownames(LM22.source) %in% rownames(addMGSM27)]
allData <- cbind(LM22.source[olGenes,], addMGSM27[olGenes,])
allData.nq <- as.data.frame(normalize.quantiles(as.matrix(allData)))
rownames(allData.nq) <- rownames(allData)
colnames(allData.nq) <- colnames(allData)
allData <- allData.nq

#Remove low variance genes to make the example run faster
allData.noNA <- allData[!apply(allData, 1, function(x){any(is.na(x))}),]
vars.noNA <- apply(allData.noNA, 1, var)

hist(vars.noNA)
```

```
#This will take a while.  To make it faster, filter genes by variance
subData <- allData[vars.noNA>2,]
gList <- rankByT(subData)
```

```
olgenes <- rownames(subData)

fullData <- allData[olgenes,colnames(LM22.source)]
colnames(fullData) <- sub('\\.[0-9]+$', '', colnames(fullData)) #Strip any trailing numbers added by make.names()

newData <- allData[olgenes,colnames(addMGSM27)]
colnames(newData) <- sub('\\.[0-9]+$', '', colnames(newData)) #Strip any trailing numbers added by make.names()

#MGSM27 <- AugmentSigMatrix(origMatrix=LM22, fullData=fullData, newData=newData, gList=gList, nGenes=1:100, plotToPDF=FALSE, imputeMissing=TRUE, condTol=1.01, postNorm=TRUE, minSumToRem=NA, addTitle=NULL, autoDetectMin=TRUE, calcSpillOver=FALSE)
#Note:  For the Identifying a High-risk Cellular Signature in the Multiple Myeloma Bone Marrow Microenvironment, we used the call commented out above, which resulted in 601 genes.  It looks like the package used to find the minimima has changed, results in a smaller number of genes (~570).  We recommend trying this option and looking at the condition number curves to see if it makes sense.  Future version will probably upgrade the 'autoDetectMin' option to be smarter. 

MGSM27 <- AugmentSigMatrix(origMatrix=LM22, fullData=fullData, newData=newData, gList=gList, nGenes=1:100, plotToPDF=TRUE, imputeMissing=TRUE, condTol=1.01, postNorm=TRUE, minSumToRem=NA, addTitle='MGSM27: ', autoDetectMin=FALSE, calcSpillOver=TRUE, pdfDir = '.')
#>   missForest iteration 1 in progress...
#> randomForest 4.6-14
#> Type rfNews() to see new features/changes/bug fixes.
#> done!
#>   missForest iteration 2 in progress...done!
#>   missForest iteration 3 in progress...done!
#>   missForest iteration 4 in progress...done!
#>   missForest iteration 5 in progress...done!
#>   missForest iteration 6 in progress...done!
#>   missForest iteration 7 in progress...done!
#>   missForest iteration 1 in progress...done!
#>   missForest iteration 2 in progress...done!
#>   missForest iteration 3 in progress...done!
#>   missForest iteration 4 in progress...done!
```

Part IV - A: Deconvolve purified CD138+ tumor samples using MGSM27

```
cellCounts <- estCellPercent.DCQ(MGSM27$sigMatrix, cd138p)
tumorPer$MGSM27.CD138p.tumor <- colSums(cellCounts[c('Plasma.cells','B.cells.memory','MM.plasma.cell','PlasmaMemory'),
    match(make.names(tumorPer$celfileRnas),colnames(cellCounts))])

incBool <- !is.na(tumorPer$CD138_Post_Sort)
curData <- tumorPer[incBool,]
rmse.mgsm27.cd138p <- sqrt(mean((curData$CD138_Post_Sort - curData$MGSM27.CD138p.tumor)^2))
ct.mgsm27.cd138p <- cor.test(curData$CD138_Post_Sort, curData$MGSM27.CD138p.tumor)
ct.mgsm27.cd138p.spear <- suppressWarnings(cor.test(curData$CD138_Post_Sort, curData$MGSM27.CD138p.tumor, method='spearman'))
titleStr <- paste0('MGSM27 Deconvolution of CD138+ Samples:\nRMSE = ', round(rmse.mgsm27.cd138p), '; Rho = ', round(ct.mgsm27.cd138p$estimate,2), '; Spear = ', round(ct.mgsm27.cd138p.spear$estimate,2) )
plot(curData$CD138_Post_Sort, curData$MGSM27.CD138p.tumor, main=titleStr, xlab='% Purity', ylab='Deconvolved Tumor %')

#Save the Deconvolution results
deconEst[['est.MGSM27.CD138p']] <- rowMeans(cellCounts, na.rm=TRUE)
```

Part IV - B: Deconvolve WBM samples using MGSM27

```
cellCounts <- estCellPercent.DCQ(MGSM27$sigMatrix, wbm)
tumorPer$MGSM27.WBM.tumor <- colSums(cellCounts[c('Plasma.cells','B.cells.memory','MM.plasma.cell','PlasmaMemory'),
    match(make.names(tumorPer$celfileRnbx),colnames(cellCounts))])

tumorPer$PC_Mean <- rowMeans(tumorPer[,c('PC_AspNum','PC_BmbxNum')], na.rm=TRUE)
incBool <- !is.nan(tumorPer$PC_Mean) & !is.na(tumorPer$MGSM27.WBM.tumor)
curData <- tumorPer[incBool,] 
rmse.mgsm27.WBM <- sqrt(mean((as.numeric(curData$PC_Mean) - curData$MGSM27.WBM.tumor)^2))
ct.mgsm27.WBM <- cor.test(as.numeric(curData$PC_Mean), curData$MGSM27.WBM.tumor)
ct.mgsm27.WBM.spear <- suppressWarnings(cor.test(as.numeric(curData$PC_Mean), curData$MGSM27.WBM.tumor, method='spearman'))
titleStr <- paste0('MGSM27 Deconvolution of WBM Samples:\nRMSE = ', round(rmse.mgsm27.WBM), '; Rho = ', round(ct.mgsm27.WBM$estimate,2), '; Spear = ', round(ct.mgsm27.WBM.spear$estimate,2) )
plot(curData$PC_Mean, curData$MGSM27.WBM.tumor, main=titleStr, xlab='% WBM Tumor', ylab='Deconvolved Tumor %')

#Save the Deconvolution results
deconEst[['est.MGSM27.WBM']] <- rowMeans(cellCounts, na.rm=TRUE)
```

Part V: Generating a new signature matrix (Top Variance, Subtract, Recreate)

```
olGenes <- rownames(LM22.source)[rownames(LM22.source) %in% rownames(addMGSM27)]
allData <- cbind(LM22.source[olGenes,], addMGSM27[olGenes,])
allData.nq <- as.data.frame(normalize.quantiles(as.matrix(allData)))
rownames(allData.nq) <- rownames(allData)
colnames(allData.nq) <- colnames(allData)
allData <- allData.nq
```

The top genes by variance will be the seedMatrix

```
nGenes <- 100

seedMat <- allData.noNA[names(tail(sort(vars.noNA), nGenes)),]
colnames(seedMat) <- sub('\\.[0-9]+$', '', colnames(seedMat))
seedMat.sig <- lapply(unique(colnames(seedMat)), function(x){ rowMeans(seedMat[,colnames(seedMat)==x], na.rm=TRUE)})
seedMat.sig <- do.call(cbind, seedMat.sig)
colnames(seedMat.sig) <- unique(colnames(seedMat))
pheatmap(seedMat.sig)
```

Augment that top gene network

```
subData.noNA <- allData.noNA[vars.noNA>2,]
colnames(allData.noNA) <- sub('\\.[0-9]+$', '', colnames(allData.noNA))
gList.noNA <- rankByT(subData.noNA)
```

```
topAug <- AugmentSigMatrix(origMatrix=seedMat.sig, fullData=allData.noNA, newData=allData.noNA, gList=gList.noNA, nGenes=1:100, plotToPDF=FALSE, imputeMissing=TRUE, condTol=1.01, postNorm=TRUE, minSumToRem=NA, addTitle=NULL, autoDetectMin=FALSE, calcSpillOver=FALSE)
```

Remove the seed genes and then rebuild

```
topAug.remSeed <- topAug[!(rownames(topAug) %in% rownames(seedMat.sig)),]

topAug2 <- AugmentSigMatrix(origMatrix=topAug.remSeed, fullData=allData.noNA, newData=allData.noNA, gList=gList.noNA, nGenes=1:100, plotToPDF=FALSE, imputeMissing=TRUE, condTol=1.01, postNorm=TRUE, minSumToRem=NA, addTitle=NULL, autoDetectMin=FALSE, calcSpillOver=FALSE)
```

Part VI - A: Deconvolve purified CD138+ tumor samples using de-novo deconvolution matrix

```
cellCounts <- estCellPercent.DCQ(topAug2, cd138p)
tumorPer$DeNovo.CD138p.tumor <- colSums(cellCounts[c('Plasma.cells','B.cells.memory','MM.plasma.cell','PlasmaMemory'),
    match(make.names(tumorPer$celfileRnas),colnames(cellCounts))])

incBool <- !is.na(tumorPer$CD138_Post_Sort)
curData <- tumorPer[incBool,]
rmse.DeNovo.cd138p <- sqrt(mean((curData$CD138_Post_Sort - curData$DeNovo.CD138p.tumor)^2))
ct.DeNovo.cd138p <- cor.test(curData$CD138_Post_Sort, curData$DeNovo.CD138p.tumor)
ct.DeNovo.cd138p.spear <- suppressWarnings(cor.test(curData$CD138_Post_Sort, curData$DeNovo.CD138p.tumor, method='spearman'))
titleStr <- paste0('DeNovo Deconvolution of CD138+ Samples:\nRMSE = ', round(rmse.DeNovo.cd138p), '; Rho = ', round(ct.DeNovo.cd138p$estimate,2), '; Spear = ', round(ct.DeNovo.cd138p.spear$estimate,2) )
plot(curData$CD138_Post_Sort, curData$DeNovo.CD138p.tumor, main=titleStr, xlab='% Purity', ylab='Deconvolved Tumor %')

#Save the Deconvolution results
deconEst[['est.dnMGSM27.CD138p']] <- rowMeans(cellCounts, na.rm=TRUE)
```

Part VI - B: Deconvolve WBM samples using de-novo deconvolution matrix

```
cellCounts <- estCellPercent.DCQ(topAug2, wbm)
tumorPer$DeNovo.WBM.tumor <- colSums(cellCounts[c('Plasma.cells','B.cells.memory','MM.plasma.cell','PlasmaMemory'),
    match(make.names(tumorPer$celfileRnbx),colnames(cellCounts))])

tumorPer$PC_Mean <- rowMeans(tumorPer[,c('PC_AspNum','PC_BmbxNum')], na.rm=TRUE)
incBool <- !is.nan(tumorPer$PC_Mean) & !is.na(tumorPer$DeNovo.WBM.tumor)
curData <- tumorPer[incBool,] 
rmse.DeNovo.WBM <- sqrt(mean((curData$PC_Mean - curData$DeNovo.WBM.tumor)^2))
ct.DeNovo.WBM <- cor.test(curData$PC_Mean, curData$DeNovo.WBM.tumor)
ct.DeNovo.WBM.spear <- suppressWarnings(cor.test(curData$PC_Mean, curData$DeNovo.WBM.tumor, method='spearman'))
titleStr <- paste0('DeNovo Deconvolution of WBM Samples:\nRMSE = ', round(rmse.DeNovo.WBM), '; Rho = ', round(ct.DeNovo.WBM$estimate,2), '; Spear = ', round(ct.DeNovo.WBM.spear$estimate,2) )
plot(curData$PC_Mean, curData$DeNovo.WBM.tumor, main=titleStr, xlab='% WBM Tumor', ylab='Deconvolved Tumor %')

#Save the Deconvolution results
deconEst[['est.dnMGSM27.WBM']] <- rowMeans(cellCounts, na.rm=TRUE)
```

Build the summary table

```
outMatrix <- matrix(0, nrow=4, ncol=4, dimnames=list(c('LM22','LM22 + 5','MGSM27','DeNovo MGSM27'),c('CD138+.RMSE','CD138+.Rho','WBM.RMSE','WBM.Rho')))
outMatrix[1,1] <- rmse.lm22.cd138p
outMatrix[1,2] <- ct.lm22.cd138p$estimate
outMatrix[1,3] <- rmse.lm22.WBM
outMatrix[1,4] <- ct.lm22.WBM$estimate

outMatrix[2,1] <- rmse.lm22p.cd138p
outMatrix[2,2] <- ct.lm22p.cd138p$estimate
outMatrix[2,3] <- rmse.lm22p.WBM
outMatrix[2,4] <- ct.lm22p.WBM$estimate
  
outMatrix[3,1] <- rmse.mgsm27.cd138p
outMatrix[3,2] <- ct.mgsm27.cd138p$estimate
outMatrix[3,3] <- rmse.mgsm27.WBM
outMatrix[3,4] <- ct.mgsm27.WBM$estimate

outMatrix[4,1] <- rmse.DeNovo.cd138p
outMatrix[4,2] <- ct.DeNovo.cd138p$estimate
outMatrix[4,3] <- rmse.DeNovo.WBM
outMatrix[4,4] <- ct.DeNovo.WBM$estimate
```

```
print(signif(outMatrix,2))
#>               CD138+.RMSE CD138+.Rho WBM.RMSE WBM.Rho
#> LM22                   27       0.26       38    0.59
#> LM22 + 5               12       0.26       37    0.53
#> MGSM27                  9       0.33       23    0.60
#> DeNovo MGSM27          36       0.18       24    0.65
```

Now print out the average deconvolution results for all cell types

```
save(deconEst, file='deconEst.ADAPTS1.RData')
allCellTypes <- unique(unlist(lapply(deconEst, function(x){names(x)})))
tumorTypes <- c('Plasma.cells','B.cells.memory','MM.plasma.cell','PlasmaMemory')

cellTypeDF <- as.data.frame(matrix(0,nrow=length(allCellTypes), ncol=length(deconEst), dimnames=list(allCellTypes, names(deconEst))))

for(curExp in names(deconEst)) {
  curDat <- deconEst[[curExp]]
  cellTypeDF[names(curDat),curExp] <- curDat
}

cellTypeDF.disp <- round(cellTypeDF, 2)

rownames(cellTypeDF.disp)[rownames(cellTypeDF.disp) %in% tumorTypes] <- paste0(rownames(cellTypeDF.disp)[rownames(cellTypeDF.disp) %in% tumorTypes], '*')


WBMexps <- colnames(cellTypeDF.disp)[grepl('WBM$', colnames(cellTypeDF.disp))]
cellDecon.WBM <- cellTypeDF.disp[,WBMexps]
print(cellDecon.WBM)
#>                              est.LM22.WBM est.LM22p5.WBM est.MGSM27.WBM
#> B.cells.naive                        0.12           0.15           0.01
#> B.cells.memory*                      0.84           0.55           0.11
#> Plasma.cells*                        9.84           4.59           4.27
#> T.cells.CD8                          1.81           1.15           0.10
#> T.cells.CD4.naive                    0.59           0.14           0.17
#> T.cells.CD4.memory.resting           0.24           0.15           0.00
#> T.cells.CD4.memory.activated         0.00           0.00           0.00
#> T.cells.follicular.helper            0.18           0.02           0.03
#> T.cells.regulatory..Tregs.           0.16           0.06           0.06
#> T.cells.gamma.delta                  3.53           4.22           2.70
#> NK.cells.resting                    11.00          11.73           5.09
#> NK.cells.activated                   1.48           1.54           0.12
#> Monocytes                           14.37          12.94           8.71
#> Macrophages.M0                       4.75           2.24           1.93
#> Macrophages.M1                       2.09           1.07           0.95
#> Macrophages.M2                       7.63           4.81           5.02
#> Dendritic.cells.resting              0.73           0.23           0.15
#> Dendritic.cells.activated            0.27           0.19           0.06
#> Mast.cells.resting                  12.11          12.40           5.23
#> Mast.cells.activated                 6.76           6.73           1.68
#> Eosinophils                          7.23           7.44           6.39
#> Neutrophils                         14.29          15.63           8.58
#> others                               0.00           0.00           0.00
#> PlasmaMemory*                        0.00           6.19          11.26
#> MM.plasma.cell*                      0.00           1.38          14.50
#> osteoclast                           0.00           2.27          13.71
#> osteoblast                           0.00           0.32           6.11
#> adipocyte                            0.00           1.86           3.08
#>                              est.dnMGSM27.WBM
#> B.cells.naive                            0.00
#> B.cells.memory*                          0.01
#> Plasma.cells*                            0.85
#> T.cells.CD8                              0.00
#> T.cells.CD4.naive                        3.99
#> T.cells.CD4.memory.resting               0.00
#> T.cells.CD4.memory.activated             0.00
#> T.cells.follicular.helper                3.65
#> T.cells.regulatory..Tregs.               3.33
#> T.cells.gamma.delta                      7.14
#> NK.cells.resting                         0.27
#> NK.cells.activated                       0.00
#> Monocytes                                4.09
#> Macrophages.M0                           0.86
#> Macrophages.M1                           1.15
#> Macrophages.M2                           4.18
#> Dendritic.cells.resting                  0.02
#> Dendritic.cells.activated                0.01
#> Mast.cells.resting                       0.00
#> Mast.cells.activated                     0.00
#> Eosinophils                             13.23
#> Neutrophils                              4.05
#> others                                   0.00
#> PlasmaMemory*                           11.51
#> MM.plasma.cell*                         16.82
#> osteoclast                              13.95
#> osteoblast                               4.54
#> adipocyte                                6.34

cd138exps <- colnames(cellTypeDF.disp)[grepl('138p$', colnames(cellTypeDF.disp))]
cellDecon.CD138p <- cellTypeDF.disp[,cd138exps]
print(cellDecon.CD138p)
#>                              est.LM22.CD138p est.LM22p5.CD138p
#> B.cells.naive                           1.76              0.92
#> B.cells.memory*                        10.38              5.27
#> Plasma.cells*                          59.59             45.49
#> T.cells.CD8                             1.06              0.13
#> T.cells.CD4.naive                       2.58              0.09
#> T.cells.CD4.memory.resting              1.33              0.13
#> T.cells.CD4.memory.activated            0.03              0.01
#> T.cells.follicular.helper               5.32              0.36
#> T.cells.regulatory..Tregs.              2.72              0.35
#> T.cells.gamma.delta                     0.03              0.03
#> NK.cells.resting                        0.11              0.09
#> NK.cells.activated                      0.03              0.01
#> Monocytes                               5.57              1.13
#> Macrophages.M0                          1.36              0.11
#> Macrophages.M1                          1.06              0.08
#> Macrophages.M2                          4.14              0.49
#> Dendritic.cells.resting                 0.95              0.11
#> Dendritic.cells.activated               0.57              0.08
#> Mast.cells.resting                      0.30              0.14
#> Mast.cells.activated                    0.06              0.03
#> Eosinophils                             0.72              0.21
#> Neutrophils                             0.32              0.29
#> others                                  0.00              0.00
#> PlasmaMemory*                           0.00             32.56
#> MM.plasma.cell*                         0.00              4.62
#> osteoclast                              0.00              0.80
#> osteoblast                              0.00              1.10
#> adipocyte                               0.00              5.37
#>                              est.MGSM27.CD138p est.dnMGSM27.CD138p
#> B.cells.naive                             0.23                0.00
#> B.cells.memory*                           2.43                0.02
#> Plasma.cells*                            32.87                7.58
#> T.cells.CD8                               0.01                0.00
#> T.cells.CD4.naive                         0.65                4.94
#> T.cells.CD4.memory.resting                0.01                0.00
#> T.cells.CD4.memory.activated              0.00                0.01
#> T.cells.follicular.helper                 1.04                4.74
#> T.cells.regulatory..Tregs.                1.13                7.37
#> T.cells.gamma.delta                       0.04                6.89
#> NK.cells.resting                          0.04                0.00
#> NK.cells.activated                        0.00                0.00
#> Monocytes                                 0.70                0.05
#> Macrophages.M0                            0.04                0.00
#> Macrophages.M1                            0.04                0.02
#> Macrophages.M2                            0.58                0.13
#> Dendritic.cells.resting                   0.07                0.00
#> Dendritic.cells.activated                 0.06                0.00
#> Mast.cells.resting                        0.02                0.09
#> Mast.cells.activated                      0.00                0.01
#> Eosinophils                               0.29                7.24
#> Neutrophils                               0.12                0.01
#> others                                    0.00                0.00
#> PlasmaMemory*                            23.67               21.76
#> MM.plasma.cell*                          33.56               29.12
#> osteoclast                                2.37                4.54
#> osteoblast                                0.01                2.93
#> adipocyte                                 0.01                2.55
```
