# Supplementary material for: ADAPTS: Automated deconvolution augmentation of profiles for tissue specific cells
Source: PLoS One. 2019 Nov 19;14(11):e0224693. doi: 10.1371/journal.pone.0224693 (PMC6863530; doi:10.1371/journal.pone.0224693)
Supplement: S2 Vig — (HTML) [file pone.0224693.s002.html]

ADAPTS Vignette #2: Single Cell Analysis


# ADAPTS Vignette #2: Single Cell Analysis

#### *Samuel Danziger, PhD*

#### *2019-06-12*

This code can install ADAPTSdata3

install.packages(‘devtools’) library(devtools) devtools::install\_github(‘sdanzige/ADAPTSdata3’)

```
library(ADAPTS)
library(ADAPTSdata3)
library(preprocessCore)
library(pheatmap)
library(foreach)
doParallel::registerDoParallel(cores = parallel::detectCores())

set.seed(42)
```

```
#Set to FALSE to use saved version of processor intensive variables
#  If set to TRUE, it will probable take 50+ minutes to complete
rebuild <- FALSE
```

Step 1: Build a signature matrix from the normal data

```
normalData <- log(ADAPTSdata3::normalData.5061+1)
```

Step 1a: Make a gList to rank genes

```
#The gList has the significantly different genes ranked by expression ratio
#  Note, this is slow
if(rebuild==TRUE) {
  gList <- ADAPTS::rankByT(normalData,remZinf = TRUE)
} else {
  gList <- ADAPTSdata3::gList
}
```

Step 1b: Determine 100 highest variance genes

```
#Find the most variant genes across cell types 
cNames <- sub('\\.+[0-9]+$','',colnames(normalData))
ctMeans <- apply(normalData, 1, function(x){tapply(x, cNames, mean, na.rm=TRUE)})

gVars <- apply(ctMeans, 2, var)
topGenes <- names(tail(sort(gVars),100))
```

Step 1c: Build the seed signature matrix & augment

```
sigMat1 <- t(ctMeans[,topGenes])

allSCdata <- normalData
colnames(allSCdata) <- cNames

topAug.var100 <- AugmentSigMatrix(origMatrix=sigMat1, fullData=allSCdata, newData=allSCdata, gList=gList, nGenes=1:100, plotToPDF=FALSE, imputeMissing=TRUE, condTol=1.01, postNorm=FALSE, minSumToRem=NA, addTitle=NULL, autoDetectMin=FALSE, calcSpillOver=TRUE)
```

Step 2: Deconvolve pseudo-bulk

```
pseudoBulk <- log(rowSums(ADAPTSdata3::normalData.5061, na.rm=TRUE)+1)
cellEst.top100 <- estCellPercent.DCQ(refExpr = sigMat1, geneExpr = data.frame(pseudoBulk=pseudoBulk))
cellEst.aug <- estCellPercent.DCQ(refExpr = topAug.var100$sigMatrix, geneExpr = data.frame(pseudoBulk=pseudoBulk))

actualFrac <- ADAPTSdata3::enumerateCellTypes(ADAPTSdata3::normalData.5061)
#> 
#>                 acinar.cell                  alpha.cell 
#>                         130                         541 
#>                   beta.cell          co.expression.cell 
#>                         152                          23 
#>                  delta.cell                 ductal.cell 
#>                          44                         179 
#>            endothelial.cell                epsilon.cell 
#>                          11                           5 
#>                  gamma.cell                   mast.cell 
#>                         107                           4 
#>           MHC.class.II.cell                    PSC.cell 
#>                           2                          32 
#> unclassified.endocrine.cell 
#>                          25
actualFrac <- actualFrac / sum(actualFrac)
actualFrac <- c(actualFrac, 0) * 100
deconTable <- data.frame(top100=cellEst.top100, augmented=cellEst.aug, ref=actualFrac)
colnames(deconTable) <- c('top100','augmented','ref')
```

Caclulate some statistics

```
AbsError <- abs(deconTable - actualFrac)
deconTableP <- as.data.frame(t(deconTable))
RMSEs <- apply(AbsError, 2, function(x){sqrt(mean(x^2))})
deconTableP$RMSE <- RMSEs
rhos <- apply(deconTable, 2, function(x){cor(x, actualFrac)})
deconTableP$rho <- rhos

deconTableP <- t(deconTableP)
colnames(deconTableP) <- c('top100', 'augmented', 'ref')
print(round(deconTableP,2))
#>                             top100 augmented   ref
#> acinar.cell                  11.38     11.56 10.36
#> alpha.cell                    7.32      7.85 43.11
#> beta.cell                     7.46      8.34 12.11
#> co.expression.cell           11.66      9.68  1.83
#> delta.cell                    7.11      7.66  3.51
#> ductal.cell                   4.36     11.49 14.26
#> endothelial.cell              0.00      2.56  0.88
#> epsilon.cell                  7.02      6.11  0.40
#> gamma.cell                    8.37      7.63  8.53
#> mast.cell                     0.00      2.13  0.32
#> MHC.class.II.cell             0.00      2.68  0.16
#> PSC.cell                      0.00      5.96  2.55
#> unclassified.endocrine.cell  35.33     16.37  1.99
#> others                        0.00      0.00  0.00
#> RMSE                         13.82     10.72  0.00
#> rho                           0.05      0.26  1.00
```

Show how combining Cell Types that are highly correlated improves correlation Step 3: Deconvolve pseudo-bulk with heirarchical deconvolution

```
#This is pretty slow

if(rebuild==TRUE) {
  hier.top100 <- ADAPTS::hierarchicalSplit(sigMatrix = sigMat1, geneExpr = allSCdata)
  hier.augment <- ADAPTS::hierarchicalSplit(sigMatrix = topAug.var100$sigMatrix, geneExpr = allSCdata)
} else {
  hier.top100 <- ADAPTSdata3::hier.top100
  hier.augment <- ADAPTSdata3::hier.augment
}
```

```
pheatmap(t(hier.top100$deconMatrices[[2]]), cluster_rows = FALSE, cluster_cols = FALSE, main='Top 100 genes:spillover matrix')

pheatmap(t(hier.top100$deconMatrices[[length(hier.top100$deconMatrices)]]), main='Top 100 genes:clustered spillover matrix')
hier.top100$allClusters
#> [[1]]
#> [1] "acinar.cell" "ductal.cell"
#> 
#> [[2]]
#> [1] "alpha.cell" "gamma.cell"
#> 
#> [[3]]
#> [1] "beta.cell"                   "co.expression.cell"         
#> [3] "delta.cell"                  "unclassified.endocrine.cell"
#> 
#> [[4]]
#> [1] "endothelial.cell" "PSC.cell"        
#> 
#> [[5]]
#> [1] "epsilon.cell"
#> 
#> [[6]]
#> [1] "mast.cell"
#> 
#> [[7]]
#> [1] "MHC.class.II.cell"

#conv <- ADAPTS::spillToConvergence(sigMatrix = sigMat1, geneExpr = allSCdata)
```

```
pheatmap(t(hier.augment$deconMatrices[[2]]), cluster_rows = FALSE, cluster_cols = FALSE, main='Augmented:spillover matrix')

pheatmap(t(hier.augment$deconMatrices[[length(hier.top100$deconMatrices)]]), main='Augmented:clustered spillover matrix')
hier.top100$allClusters
#> [[1]]
#> [1] "acinar.cell" "ductal.cell"
#> 
#> [[2]]
#> [1] "alpha.cell" "gamma.cell"
#> 
#> [[3]]
#> [1] "beta.cell"                   "co.expression.cell"         
#> [3] "delta.cell"                  "unclassified.endocrine.cell"
#> 
#> [[4]]
#> [1] "endothelial.cell" "PSC.cell"        
#> 
#> [[5]]
#> [1] "epsilon.cell"
#> 
#> [[6]]
#> [1] "mast.cell"
#> 
#> [[7]]
#> [1] "MHC.class.II.cell"

#conv <- ADAPTS::spillToConvergence(sigMatrix = sigMat1, geneExpr = allSCdata)
```

```
groups <- sapply (unlist(hier.top100$allClusters), function(g) {
  which(sapply(hier.top100$allClusters, function(x){g %in% x}))
})
groups <- c(groups, others=max(groups)+1)

comb.top100 <- apply(deconTable, 2, function(x){tapply(x, groups, sum)})
cors.top100 <- apply(comb.top100, 2, function(x){cor(x, comb.top100[,'ref'])})
rmses.top100 <- apply(comb.top100, 2, function(x){sqrt(mean((x-comb.top100[,'ref'])^2))})

combP.top100 <- as.data.frame(t(comb.top100))
colnames(combP.top100) <- c(sapply(hier.top100$allClusters, function(x){paste(x, collapse='_')}),'others')

combP.top100$RMSE <- rmses.top100
combP.top100$rho <- cors.top100

combP.top100 <- t(combP.top100)
combP.top100 <- combP.top100[,c(1,3)]

print('Top 100 Combination Predictions')
#> [1] "Top 100 Combination Predictions"
print(round(combP.top100,2))
#>                                                                     top100
#> acinar.cell_ductal.cell                                              18.70
#> alpha.cell_gamma.cell                                                19.12
#> beta.cell_co.expression.cell_delta.cell_unclassified.endocrine.cell  18.49
#> endothelial.cell_PSC.cell                                             8.37
#> epsilon.cell                                                          0.00
#> mast.cell                                                             0.00
#> MHC.class.II.cell                                                    35.33
#> others                                                                0.00
#> RMSE                                                                 17.15
#> rho                                                                   0.32
#>                                                                       ref
#> acinar.cell_ductal.cell                                             53.47
#> alpha.cell_gamma.cell                                               13.94
#> beta.cell_co.expression.cell_delta.cell_unclassified.endocrine.cell 19.04
#> endothelial.cell_PSC.cell                                            8.84
#> epsilon.cell                                                         0.16
#> mast.cell                                                            2.55
#> MHC.class.II.cell                                                    1.99
#> others                                                               0.00
#> RMSE                                                                 0.00
#> rho                                                                  1.00
```

```
groups <- sapply (unlist(hier.augment$allClusters), function(g) {
  which(sapply(hier.augment$allClusters, function(x){g %in% x}))
})
groups <- c(groups, others=max(groups)+1)

comb.augment <- apply(deconTable, 2, function(x){tapply(x, groups, sum)})
cors.augment <- apply(comb.augment, 2, function(x){cor(x, comb.augment[,'ref'])})
rmses.augment <- apply(comb.augment, 2, function(x){sqrt(mean((x-comb.augment[,'ref'])^2))})

combP.augment <- as.data.frame(t(comb.augment))
colnames(combP.augment) <- c(sapply(hier.augment$allClusters, function(x){paste(x, collapse='_')}),'others')

combP.augment$RMSE <- rmses.augment
combP.augment$rho <- cors.augment

combP.augment <- t(combP.augment)
combP.augment <- combP.augment[,c(2,3)]

print('Augment Combination Predictions')
#> [1] "Augment Combination Predictions"
print(round(combP.augment,2))
#>                                                                                           augmented
#> acinar.cell_ductal.cell                                                                       19.41
#> alpha.cell_beta.cell_co.expression.cell_delta.cell_gamma.cell_unclassified.endocrine.cell     45.84
#> endothelial.cell_PSC.cell                                                                      9.76
#> epsilon.cell                                                                                   2.68
#> mast.cell_MHC.class.II.cell                                                                   22.33
#> others                                                                                         0.00
#> RMSE                                                                                          16.58
#> rho                                                                                            0.58
#>                                                                                             ref
#> acinar.cell_ductal.cell                                                                   53.47
#> alpha.cell_beta.cell_co.expression.cell_delta.cell_gamma.cell_unclassified.endocrine.cell 32.99
#> endothelial.cell_PSC.cell                                                                  8.84
#> epsilon.cell                                                                               0.16
#> mast.cell_MHC.class.II.cell                                                                4.54
#> others                                                                                     0.00
#> RMSE                                                                                       0.00
#> rho                                                                                        1.00
```

Step 4: Hierarchical Deconvolution

```
cellEst.top100.hier <- ADAPTS::hierarchicalClassify(sigMatrix = sigMat1, toPred = data.frame(pseudoBulk=pseudoBulk), geneExpr = allSCdata, hierarchData = hier.top100)
#>   missForest iteration 1 in progress...done!
#>   missForest iteration 2 in progress...done!
#>   missForest iteration 1 in progress...done!
#>   missForest iteration 2 in progress...done!
#>   missForest iteration 1 in progress...done!
#>   missForest iteration 2 in progress...done!
#>   missForest iteration 1 in progress...done!
#>   missForest iteration 2 in progress...done!
#>   missForest iteration 1 in progress...done!
#>   missForest iteration 2 in progress...done!
cellEst.top100.hier
#>                             pseudoBulk
#> acinar.cell                   7.884952
#> ductal.cell                   7.853475
#> alpha.cell                    7.922658
#> gamma.cell                    7.765773
#> beta.cell                    11.745648
#> co.expression.cell           16.910532
#> delta.cell                   12.268908
#> unclassified.endocrine.cell  20.628756
#> endothelial.cell              0.000000
#> PSC.cell                      0.000000
#> epsilon.cell                  7.019298
#> mast.cell                     0.000000
#> MHC.class.II.cell             0.000000
#> others                        0.000000
```

```
cellEst.augment.hier <- ADAPTS::hierarchicalClassify(sigMatrix = topAug.var100$sigMatrix, toPred = data.frame(pseudoBulk=pseudoBulk), geneExpr = allSCdata, hierarchData = hier.augment)
#>   missForest iteration 1 in progress...done!
#>   missForest iteration 2 in progress...done!
#>   missForest iteration 1 in progress...done!
#>   missForest iteration 2 in progress...done!
#>   missForest iteration 1 in progress...done!
#>   missForest iteration 2 in progress...done!
#>   missForest iteration 1 in progress...done!
#>   missForest iteration 2 in progress...done!
#>   missForest iteration 1 in progress...done!
#>   missForest iteration 2 in progress...done!
cellEst.augment.hier
#>                             pseudoBulk
#> acinar.cell                 10.4856529
#> ductal.cell                 12.5597381
#> alpha.cell                   9.5135593
#> beta.cell                    8.5069856
#> co.expression.cell          10.7674625
#> delta.cell                   8.4092042
#> gamma.cell                   8.0353339
#> unclassified.endocrine.cell 12.2859508
#> endothelial.cell             0.0000000
#> PSC.cell                     8.5182963
#> epsilon.cell                 6.1087782
#> mast.cell                    0.7992621
#> MHC.class.II.cell            4.0097760
#> others                       0.0000000
```

Combine and calculate statistics

```
deconTable.hier <- data.frame(top100.hier=cellEst.top100.hier, augmented.hier=cellEst.augment.hier)
colnames(deconTable.hier) <- c('top100.hier','augmented.hier')
deconTable.all <- cbind(deconTable.hier, deconTable)
deconTableP.all <- as.data.frame(t(deconTable.all))

#AbsError.all <- abs(deconTable.all - actualFrac)
#RMSEs.all <- apply(AbsError.all, 2, function(x){sqrt(mean(x^2))})
#rhos.all <- apply(deconTable.all, 2, function(x){cor(x, actualFrac)})

cors.aug <- apply(deconTable.all, 2, function(x){cor(x, deconTable.all[,'ref'])})
rmses.aug <- apply(deconTable.all, 2, function(x){sqrt(mean((x-deconTable.all[,'ref'])^2))})

deconTableP.all$RMSE <- rmses.aug
deconTableP.all$rho <- cors.aug

deconTableP.all <- t(deconTableP.all)
#colnames(deconTableP) <- c('top100', 'augmented', 'ref')
deconTableP.all <- deconTableP.all[,c('top100','top100.hier','augmented','augmented.hier','ref')]
colnames(deconTableP.all) <- c('top','top.hier','aug','aug.hier','ref')
print('Normal Cell Predictions')
#> [1] "Normal Cell Predictions"
print(round(deconTableP.all,2))
#>                               top top.hier   aug aug.hier   ref
#> acinar.cell                 11.38     7.88 11.56    10.49 10.36
#> ductal.cell                  7.32     7.85  7.85    12.56 43.11
#> alpha.cell                   7.46     7.92  8.34     9.51 12.11
#> gamma.cell                  11.66     7.77  9.68     8.51  1.83
#> beta.cell                    7.11    11.75  7.66    10.77  3.51
#> co.expression.cell           4.36    16.91 11.49     8.41 14.26
#> delta.cell                   0.00    12.27  2.56     8.04  0.88
#> unclassified.endocrine.cell  7.02    20.63  6.11    12.29  0.40
#> endothelial.cell             8.37     0.00  7.63     0.00  8.53
#> PSC.cell                     0.00     0.00  2.13     8.52  0.32
#> epsilon.cell                 0.00     7.02  2.68     6.11  0.16
#> mast.cell                    0.00     0.00  5.96     0.80  2.55
#> MHC.class.II.cell           35.33     0.00 16.37     4.01  1.99
#> others                       0.00     0.00  0.00     0.00  0.00
#> RMSE                        13.82    12.09 10.72    10.16  0.00
#> rho                          0.05     0.12  0.26     0.39  1.00
```

Step 5: Deconvolve Diabetes data

```
pseudoBulk.diabetes <- log(rowSums(ADAPTSdata3::diabetesData.5061, na.rm=TRUE)+1)
pb.d.df <- data.frame(pseudoBulk.diabetes=pseudoBulk.diabetes)
cellEst.d.top100 <- estCellPercent.DCQ(refExpr = sigMat1, geneExpr = pb.d.df)
cellEst.d.augment <- estCellPercent.DCQ(refExpr = topAug.var100$sigMatrix, geneExpr = pb.d.df)


actualFrac.diabetes <- ADAPTSdata3::enumerateCellTypes(ADAPTSdata3::diabetesData.5061)
#> 
#>                 acinar.cell                  alpha.cell 
#>                          55                         345 
#>                   beta.cell          co.expression.cell 
#>                         118                          16 
#>                  delta.cell                 ductal.cell 
#>                          70                         207 
#>            endothelial.cell                epsilon.cell 
#>                           5                           2 
#>                  gamma.cell                   mast.cell 
#>                          90                           3 
#>           MHC.class.II.cell                    PSC.cell 
#>                           3                          22 
#> unclassified.endocrine.cell 
#>                          16
actualFrac.diabetes <- actualFrac.diabetes / sum(actualFrac.diabetes)
actualFrac.diabetes <- c(actualFrac.diabetes, 0) * 100

#Heirarchical Deconvolution
cellEst.d.top100.hier <- ADAPTS::hierarchicalClassify(sigMatrix = sigMat1, toPred = pb.d.df, geneExpr = allSCdata, hierarchData = hier.top100)
#>   missForest iteration 1 in progress...done!
#>   missForest iteration 2 in progress...done!
#>   missForest iteration 1 in progress...done!
#>   missForest iteration 2 in progress...done!
#>   missForest iteration 1 in progress...done!
#>   missForest iteration 2 in progress...done!
#>   missForest iteration 1 in progress...done!
#>   missForest iteration 2 in progress...done!
#>   missForest iteration 1 in progress...done!
#>   missForest iteration 2 in progress...done!
cellEst.d.augment.hier <- ADAPTS::hierarchicalClassify(sigMatrix = topAug.var100$sigMatrix, toPred = pb.d.df, geneExpr = allSCdata, hierarchData = hier.augment)
#>   missForest iteration 1 in progress...done!
#>   missForest iteration 2 in progress...done!
#>   missForest iteration 1 in progress...done!
#>   missForest iteration 2 in progress...done!
#>   missForest iteration 1 in progress...done!
#>   missForest iteration 2 in progress...done!
#>   missForest iteration 1 in progress...done!
#>   missForest iteration 2 in progress...done!
#>   missForest iteration 1 in progress...done!
#>   missForest iteration 2 in progress...done!
```

```
deconTable.d.aug <- data.frame(top100.d=cellEst.d.top100, top100.d.hier=cellEst.d.top100.hier, augmented.d=cellEst.d.augment, augmented.d.hier=cellEst.d.augment.hier, ref.d=actualFrac.diabetes)
colnames(deconTable.d.aug) <- c('top.d', 'top.d.hier', 'aug.d', 'aug.d.hier', 'ref.d')

cors.d.aug <- apply(deconTable.d.aug, 2, function(x){cor(x, deconTable.d.aug[,'ref.d'])})
rmses.d.aug <- apply(deconTable.d.aug, 2, function(x){sqrt(mean((x-deconTable.d.aug[,'ref.d'])^2))})

deconTableP.d.aug <- as.data.frame(t(deconTable.d.aug))
deconTableP.d.aug$RMSE <- rmses.d.aug
deconTableP.d.aug$rho <- cors.d.aug

print('Deconvolution of diabetes samples')
#> [1] "Deconvolution of diabetes samples"
print(round(t(deconTableP.d.aug),2))
#>                             top.d top.d.hier aug.d aug.d.hier ref.d
#> acinar.cell                  7.40       4.32 10.82       8.89  5.78
#> alpha.cell                   7.93       9.01  7.94      14.41 36.24
#> beta.cell                    8.04       8.44  8.58       9.35 12.39
#> co.expression.cell          12.33       7.95 10.03       8.55  1.68
#> delta.cell                   9.38      11.50  8.13      10.93  7.35
#> ductal.cell                  5.93      17.06 12.49       8.90 21.74
#> endothelial.cell             0.00      13.80  2.58       7.91  0.53
#> epsilon.cell                 6.56      21.36  5.83      12.12  0.21
#> gamma.cell                   8.46       0.00  7.61       0.00  9.45
#> mast.cell                    0.00       0.00  1.72       8.51  0.32
#> MHC.class.II.cell            0.00       6.56  2.88       5.83  0.32
#> PSC.cell                     0.00       0.00  5.93       0.55  2.31
#> unclassified.endocrine.cell 33.97       0.00 15.47       4.05  1.68
#> others                       0.00       0.00  0.00       0.00  0.00
#> RMSE                        12.76      10.68  9.44       8.91  0.00
#> rho                          0.06       0.24  0.35       0.46  1.00
```
